# Frequency Based Design Partitioning to Achieve Higher Throughput in Digital Cross Correlator for Aperture Synthesis Passive MMW Imager

**DOI:** 10.3390/s18041238

**Published:** 2018-04-17

**Authors:** Muhammad Asif, Xiangzhou Guo, Jing Zhang, Jungang Miao

**Affiliations:** School of Electronic Information Engineering, Beihang University, No. 37, Xueyuan Road, Haidian District, Beijing 100191, China; guo_xiangzhou@hotmail.com (X.G.); yinizj@163.com (J.Z.); jmiaobremen@buaa.edu.cn (J.M.)

**Keywords:** FPGA, digital correlator, radiometer, passive imager, aperture synthesis

## Abstract

Digital cross-correlation is central to many applications including but not limited to Digital Image Processing, Satellite Navigation and Remote Sensing. With recent advancements in digital technology, the computational demands of such applications have increased enormously. In this paper we are presenting a high throughput digital cross correlator, capable of processing 1-bit digitized stream, at the rate of up to 2 GHz, simultaneously on 64 channels i.e., approximately 4 Trillion correlation and accumulation operations per second. In order to achieve higher throughput, we have focused on frequency based partitioning of our design and tried to minimize and localize high frequency operations. This correlator is designed for a Passive Millimeter Wave Imager intended for the detection of contraband items concealed on human body. The goals are to increase the system bandwidth, achieve video rate imaging, improve sensitivity and reduce the size. Design methodology is detailed in subsequent sections, elaborating the techniques enabling high throughput. The design is verified for Xilinx Kintex UltraScale device in simulation and the implementation results are given in terms of device utilization and power consumption estimates. Our results show considerable improvements in throughput as compared to our baseline design, while the correlator successfully meets the functional requirements.

## 1. Introduction

Aperture synthesis using interferometric technique has long been used for synthesizing very large apertures for radio telescopes like Very Large Array (VLA), Atacama Large Millimeter/sub-millimeter Array (ALMA) and Allen Telescope Array (ATA). It has successfully been used for remote sensing mission with all-weather capabilities, Soil moisture and Ocean Salinity (SMOS) being the first satellite carrying such payload while Geostationary Synthetic Thinned Aperture Radiometer (GeoSTAR) and Geostationary Atmospheric Sounder (GAS) are also being developed using the similar imaging technique. In recent past, Synthetic Aperture Interferometric Radiometry (SAIR) has been found useful for a few other applications, being one of them is passive millimeter wave imaging for detection of concealed dangerous/prohibited items [1].

SAIR based passive MMW security screening systems present a very effective option for maintaining security and controlling illegal trafficking as they are sensitive to not only metallic objects but to non-metallic items as well and can provide video rate imaging with large field of view. Passive imaging technique make them safer as the persons or articles being scanned are not subjected to any radiations. These imagers calculate cross-correlation between all the possible pairs of baseband signals after quadrature demodulation, to form a visibility function sample [2]. Since the signal to noise ratio (SNR) of passive imagers is very low, usually the cross-correlation results need to be integrated over a long period of time [3]. As given in basic radiometer equation (1), the SNR is proportional to the square root of the product of bandwidth and integration time [4]. Thus, we can also improve the SNR by increasing bandwidth despite a relatively short time available for a security scanner equipment to observe a moving person/object.

(1) $$R_{SN}\;=\;C\;\frac{T_{A}}{T_{S}}\sqrt{∆\nu \;\tau }$$

where,

- $R_{SN}$ = Signal to Noise Ratio
- C = Constant greater than or equal to 1
- $T_{A}$ = Antenna Temperature
- $T_{S}$ = System Temperature
- ∆ν = Bandwidth
- τ = Signal Averaging Time

**Figure 1:**
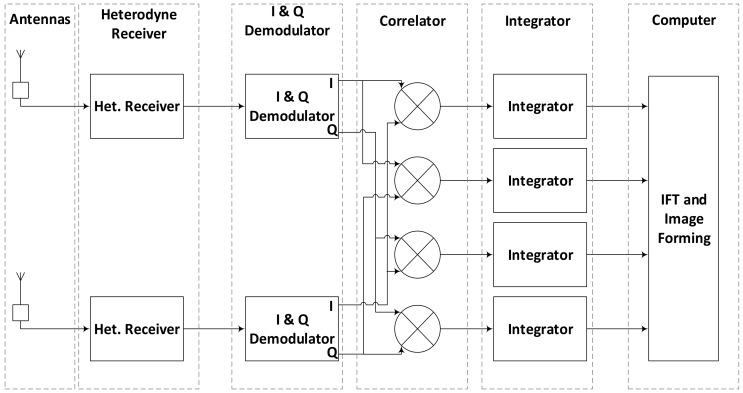
2 Antennas based complete Synthetic Aperture Interferometric Radiometry (SAIR) Imager.

The data sampling rate is, therefore, important for such passive imaging systems to improve SNR and imaging rate. The brightness temperature image is finally formed by applying inverse Fourier transform to the time integrated visibility function samples as per Van-Cittert-Zernike theorem. A complete flow of operations in SAIR imaging system is shown in Figure 1 while theory of the radiometric imaging is discussed in detail in [3,4,5,6,7,8,9].

Two versions of Passive Millimeter Wave Imagers for security screening application i.e., BHU-2D [5] and BHU-2D-U [3] have already been developed and tested by Beihang University. BHU-2D have 24 receiving elements with 160 MHz bandwidth and BHU-2D-U have 48 receiving elements with 200 MHz bandwidth [3]. The work being presented in this article is a part of further enhancements in this system targeted to improve sensitivity and increase resolution in video rate imaging. The new imaging system will use an increased number of antennas to achieve these objectives i.e., approximately 1024, with bandwidth greater than 1 GHz in W band, just like suggested in [10] for their TRL6 prototype. Considering the required amount of processing for a 2-D aperture synthesis imager in the context of current state of the art digital technology, it is challenging to find a feasible solution. We are, therefore, proceeding with two-pronged strategy; 1st is 2-D aperture synthesis with thinned array having high imaging rate similarly as presented in [11] & 2nd is the combination of aperture synthesis with analog phased-array (aperture synthesis in one dimension and analog phased-array in the other) with full filled array which is expected to have relatively low imaging rate for the similar level of sensitivity and resolution.

### Related Work

Ever since the first digital correlator realized by Weinreb in 1963 [12], its complexity and capabilities have increased many folds. Most of the recent, new generation radio telescope arrays are equipped with very complex and powerful correlators, where major processing tasks are carried out using state of the art Field Programmable Gate Arrays (FPGAs) [13], Graphics Processing Units (GPUs) [14], Application Specific Integrated Circuits (ASICs) [15,16] or even a combination of these processing elements. The Expanded Very Large Array, for example, is equipped with WIDAR correlator consisting 256 large sized (38 cm × 48 cm), 28-layer printed circuit boards [17]. Such large, expensive and high power consuming systems are, obviously, not suitable for security screening application. On the other hand, air-borne or space-borne radiometers are relatively less complex and are designed for space and power efficiency. These features make them look similar to security screening imaging systems. Therefore, we focus mainly on such systems for our literature review in the next few paragraphs and results comparison in general.

Erik Ryman et al. in [18], presented a 64-input correlator ASIC capable of performing correlations and integration maximum at the rate of 3.6 GHz while dissipating only 790 mW of power. It implements zero-lag correlations only and performs a total of 2016 correlations and integrations for each tick of sampling clock. This project uses 1-bit digitization which is performed by another specially designed comparator ASIC [19]. The system architecture, is quite efficient with two stage integration. First stage comprises of 6-bit integrator while higher 24 bits are implemented as ripple counter making it 30-bit integration capable of integrating 0.5 s of data at 2 GHz sampling speed.

The correlator developed in the University of Michigan for GeoSTAR project is another example of similar work. The first prototype was developed using an FPGA implementing 1-bit correlator working at the 100 MHz [8,20]. Due to high power consumption and limited bandwidth [21] that could be achieved using an FPGA, the next effort was targeted to develop an ASIC for this job. This chip developed for GeoSTAR-II was able to perform 19 × 19, 2-bit correlations with 500 MHz bandwidth while consuming 250 uW/correlation. For the next prototype i.e., GeoSTAR-III a 64 × 64 correlator ASIC was developed with integrated 2-bit A/D conversion for 64 inputs channels and could operate at a nominal frequency of 1 GHz [22].

The system configuration of the L-band synthetic aperture radiometer, developed by Helsinki University of Technology, presented in [7] is quite similar to the work being presented in this article. This instrument uses 1-bit digitization at a nominal sampling frequency of 60 MHz. The correlator circuit implemented in FPGA is comprised of 2704 correlators that can integrate the correlation results up to 4 s. The implementation of correlator is carried out in three stages i.e., a 4-bit pre-scaler stage and a 6-bit integrator before using internal RAM for a total of 24-bit accumulation. The whole sequence of operations is defined in two sub-cycles. First cycle includes correlation, 4-bit pre-scaler and 6-bit integrator while the second sub-cycle includes the accumulation of all the results from first sub-cycle i.e., the outputs of all the integrators using FPGA’s internal block RAM.

## 2. Digital Signal Processing Requirements

For the digitization of an analog signal, there exist many possibilities ranging from 1 to several bits quantization. It has been reported in [23] that 2-level digitization is quite noisy and least efficient, whereas much of this loss in efficiency can be recovered by 3-level A/D conversion. Going beyond that i.e., 4-level or more, however, results in minor improvements only. 2B/3L digitization, therefore, is efficient (ɳ_Q_ = 0.8) and appears to be the best trade-off [23], at least on paper in ideal case. On the other hand, when taking into consideration the practical issues of higher communication bandwidth, system complexity and hardware utilization, 1B/2L digitization scheme proves to be a better option, especially when the number of input channels is large and bandwidth is high. 1B/2L scheme has the potential to provide an optimum solution in all aspects at the cost of minimal loss of efficiency [24]. This approach is already proving good in many practical situations, for example systems referred in [7,8]. Therefore, we have adopted 1B/2L A/D conversion for our system [25].

The instruments designed for continuum observation are required to measure an average correlation over the entire bandwidth of the signal [4,19], whereas those for spectral lines analysis may require to analyze different frequency components across multiple sub-bands of the entire bandwidth by calculating correlations between several lags of each input. Since an imaging system like proposed in this work falls in the category of continuum observation instruments, we rely only on zero offset signals and implement our correlator in XF configuration for being more intuitive and its simplistic development.

The bottle neck in implementation of a SAIR system lies in the movement of the data from several channels and processing it in parallel in the order of hundreds of MHz or even in GHz. Correlation and integration operations are the most compute intensive parts as compared to the other operations in a SAIR imager shown in Figure 1. The number of operations is given as

(2) $$n_{Op}\;=\;\left(2n_{a}^{2}\;-\;n_{a}\right)\;F_{S}$$

where,

- $n_{Op}$ = No. of Operations
- $n_{a}$ = No. of Antennas
- $F_{S}$ = Sampling Frequency

Thus, if we go for a 2-D aperture synthesis system, it will increase the throughput and processing requirements of digital backend system, enormously i.e., in the order of 10^15^ operations per second, for a 1024 antennas system having bandwidth equal to 1 GHz.

Obviously, performing this amount of processing in real time can result in a very complex and costly system which would not be suitable for security application. Therefore, as mentioned above, we have adopted a two-pronged strategy and it has been decided to find an efficient architecture for correlator to process the outputs from 32 antennas at first step and then build upon this design to achieve the capability of processing 1024 antennas, at the next level of hierarchy. For 2-D aperture synthesis approach, the future course of action is largely dependent upon how many channels of correlator can be accommodated in a single device. While for the second approach, this design is planned to be used “as it is” for the first prototype. For a 32-antenna correlator, if (32I + 32Q) 64 signals are sampled at 2 GHz, our basic correlator module will require around 4 trillion correlation and accumulation operations per second. At the time of this writing, such performance is only reported by a few dedicated correlator ASICs whereas the goal of work being presented here is to achieve this performance in an FPGA chip with enough room left for A/D interface and control logic. It would result in a single chip digital correlation solution for 32 antennas and help build more complex imaging system using multiple FPGA chips by reducing the complexity in next level of hierarchy.

## 3. Correlation System Design

In this section we describe our design and the design logic from all aspects.

### 3.1. Implementation Platform

Generally, most of the recent high-performance, real-time digital processing systems are developed using FPGA, GPU or ASIC depending upon the performance requirements and nature of application. GPUs are very powerful data processing devices that can process a lot of data in parallel and are very effective for DSP operations on multiple parallel data streams even in real time. However, they have their own fixed architecture, data flow and programming requirements with limited I/O capabilities. GPUs cannot directly perform real-time data acquisition, requiring another device to interface with A/D chips. They are most often used for implementing complex DSP algorithms with integer or floating-point operands. Using such devices for simply correlating and integrating a few bit operands might not utilize their strength optimally. There are a few examples of using GPUs in large radio telescope systems such as Murchison Widefield Array (MWA) [14], but even in this system data acquisition and correlation is performed by FPGA devices and GPUs are only used for carrying out some complex processing tasks after correlation. ASIC, on the other hand, provides the most efficient and customizable solution as it can also be observed that two of the most recent relevant projects i.e., GAS [19] and GeoSTAR [22] chose to go for ASIC as the best possible solution. However, considering its high Non-Recurring Engineering (NRE) cost limits this option for our project at this stage when we are still trying different techniques for obtaining the best results. In this situation, FPGAs are the optimum solution providing a lot of customization opportunities within an affordable budget. Hence we chose Xilinx Kintex UltraScale device (XCKU115) for our system being moderate to high cost device offering plenty of resources that can accommodate quite a handsome amount of functionality.

### 3.2. Design Partition

In order to achieve high throughput and enable efficient routing, we have partitioned our design into high-speed and low-speed parts; low-speed clock being 6 times slower than the high-speed clock. Both, the high-speed and the low-speed clocks are derived from the same clock so as to avoid the problems emerging from asynchronous clock domain crossing. The integration is divided into two stages just like [7,19]. The high-speed circuit is localized only within correlator and first stage integration module. The difference from [7,19] is that they implemented the first stage as a pre-scaler which is not a part of readout values, while we have implemented it as two stage accumulation. The idea of design partition in low-speed and high speed clock domains arisen from our baseline implementation [26] where all the correlation and accumulation operations are carried out on the same, high-speed clock. It was observed that all the critical paths existed between the first stage integrator and the second stage, where signals from 256 first stage integrators were being accumulated by a pair of an adder and a DPRAM. This is quite obvious with our system’s architecture that divides the three operational steps into two stages or sub-cycles where each channel has dedicated hardware resources for first stage while the hardware for second stage is shared between 256 channels. As a result, the hardware for first stage can be implemented in a confined place with shorter data paths while the hardware for second stage has 256 multi-bit data paths that must be implemented relatively at distant places in a device. Two options were considered to address this problem; first was to introduce a pipeline stage between both the stages and the second was partitioning the design into high-speed and low-speed clock domains. Both the options involved adding some hardware but adding pipeline stage for multi-bit data lines is more expensive than adding just a few flip-flops for control signals. Thus, the second option was preferred, in addition, it also had the potential to decrease the power consumption. Gains and costs of this technique can be seen in results section.

**Figure 2:**
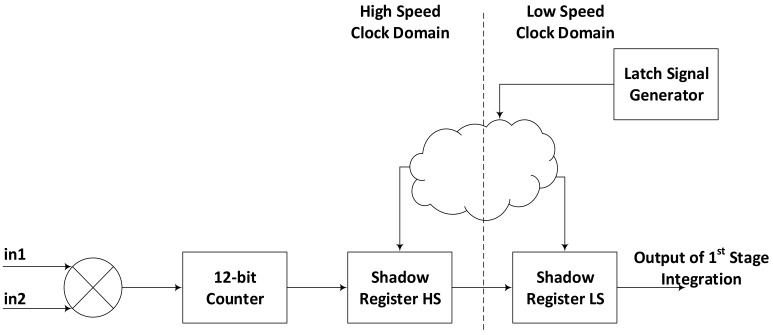
Correlation and first stage integration with high-speed and low-speed clock domains.

The control signals entering the correlator and first stage integrator, for latching the results of first stage integration are referenced with slower clock whereas the inputs are obviously referenced with high-speed clock. Control signals are then shifted to high-speed clock domain where all the correlation and first stage integration is performed. As the control signal for data latching is asserted, the results of first stage integration are latched in high-speed clock domain. The latched values are then shifted to slower clock domain for further processing in the second stage. In this way all the signals and values that might have to travel long paths between the modules implemented at some distances get more time for register-to-register movement while signals confined within a small area i.e., within correlator and first stage integrator are referenced with high speed clock. This partition in indicated in Figure 2.

### 3.3. First Stage Integration

Complete correlation and integration is divided into three steps. In first step, the correlation between two 1-bit inputs is calculated with XNOR operation. In the second step, each correlation result controls the operation of an up-counter, used as first stage integrator. The counter value is stored in a dual latch each time the latch signal is asserted. The dual latch consists of two consecutive shadow registers; first one is clocked with high-speed clock while second register is driven by slower clock. Figure 2 shows the complete picture of hardware for correlation and first stage integration. Since, for 64 channel correlator, there are 2016 possible input combinations and hence the number of correlation and first stage integration units, we opted to design this part in FPGA fabric using logic resources instead of dedicated DSP48 slices. Although in XCKU115 device, we can afford to use DSP slices but that would have made our design too much device dependent as this many DSP slices are available only in the top devices of any FPGA family. Secondly, these DSP slices are distributed over the device, using them would have caused unnecessarily long routes as well. Thirdly, if we would have to expand this design for more than 64 channels, the number of possible input combinations would go beyond the available DSP slices in any FPGA device. After first stage integration, second stage integration is started.

### 3.4. Second Stage Integration

**Figure 3:**
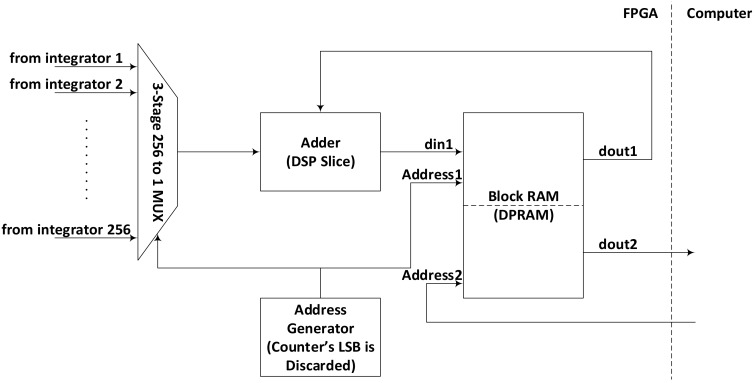
Second stage of integration.

While first stage integration is implemented using dedicated hardware for each pair of correlation, the second stage uses a multiplexed approach by integrating results from 256 first stage integrators in one second stage integrator, thus requiring 8 such modules for 2016 pairs. There is a 10-bit counter operating at slower clock, which marks the start of second stage integration cycle with each overflow. It is shown in Figure 2 as latch signal generator. As soon as the start of second stage integration is indicated by latch signal generator, the output of first stage integration is latched into a register and moved subsequently to another register referenced with the slower clock. The second stage integration is performed by the combination of a DPRAM and a DSP slice, configuration of which is explained in Figure 3. Out of possible configurations of internal RAM available in FPGA, 512 × 36 is found to be the most suitable for this system, although there are other configurations offering different combinations of width and depth. Reducing width cannot provide us the required integration and increasing width simply exceeds the integration requirements. Similarly increasing the length of the DPRAM can increase the number of sequentially performed integration operations, causing more cycles to finish the second integration cycle while reducing the length can results in inefficient use of RAM blocks that can cause increased power consumption.

This 512 × 36 DPRAM is used in ping-pong configuration, with only 256 locations being used at a time while sparing the other 256 for computer read operation. Whenever, the master computer asserts that a read operation is going to begin, the second stage integration switches to the other half of the DPRAM for further operation. This way, the system keeps integrating and computer keeps reading the values, at the same time and without interfering each other. It helps maintaining coherence of the data read by the computer by presenting a picture where all the results belong to the same instance of time.

Another counter clocked by the slower clock is used as address generator for the second stage integrator. The second stage integration involves reading a particular location in DPRAM, adding a corresponding first stage integration value with it using a DSP slice and finally writing it back to the same location. The DPRAM requires one clock cycles to present the data at its output after read is enabled with a valid address at its input and at least one clock cycle time is required for add operation in DSP slice. The whole accumulation/integration process requires two clock cycles for its safe operation. We, therefore, dedicate two clock cycles for each integration operation by discarding the lowest significant bit of the address generator and using only upper 8 bits for addressing. This way it takes 512 clock cycles to complete second stage integration cycle. 

### 3.5. Readout

As the master computer asserts signal for start of read cycle, that particular half of the DPRAM being used at that instance of time for second stage integration is spared for computer readout and the alternate half is dedicated for the next cycle of second stage integration. However, if an integration process is already in progress, then this switching is delayed to let the cycle complete. Since the read operation is supported by maximum at slower clock speed and we use the RAM in dual port configuration, computer can safely read while second stage integration process is writing at some other location in the same half.

### 3.6. Parallel Operation

**Figure 4:**
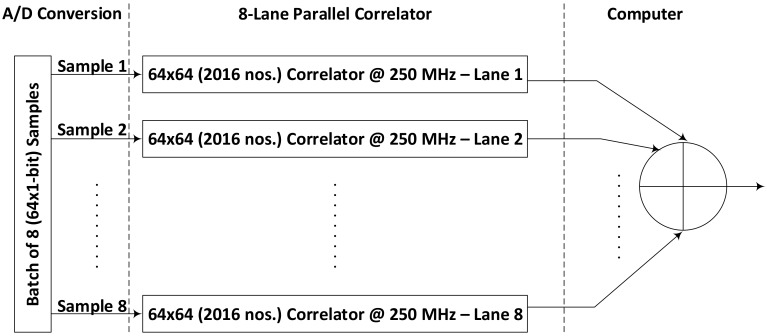
Achieving 2 GHz throughput from 8-lane parallel operation of correlator working at 250 MHz.

We assume that the correlator receives the input data digitized at a maximum speed of 2 GHz, but the FPGA technology, due to its flexibility and configurable nature cannot support such high-speed operations. This fact limits the bandwidth achievable on FPGA devices [21]. Since we are using the largest available device in Kintex UltraScale family offering abundant on-chip resources, we introduced some parallel processing in our design. Figure 4 shows how eight parallel processing correlators are used to enable the processing of 8 samples at a time as suggested by [27] (Here, 64 1-bit samples from I & Q channels of 32 antennas sampled at an instance of time is referred to as one sample).

It is kind of a batch processing idea. The input data streams from all the channels are accumulated for 8 cycles and then this batch of data is forwarded to 8 correlator channels. In this way, each correlator correlates and integrates the incoming data samples with a gap of 7 samples. Correlator 1, for example, correlates the data samples 1, 9, 17 and so on while correlator 2 process the data samples 2, 10, 18 and so on. The master computer reads the results from these 8 parallel operating correlators and can add their results for each channel to make a full integration result. Using these parallel datapaths, we managed to process the data sampled at 2 GHz by running this 8-lane parallel processing correlator unit at 250 MHz.

## 4. Design Verification and Results

The design is verified for its functional correctness in simulation using Vivado Simulator. A set of pseudo-random data consisting 65 K samples for each of 64 channels, was generated in MATLAB. It served as the digitized input stream for correlator. A code in MATLAB was developed to calculate the correlation of this data set and integrate it for the whole length, just like it is expected of our FPGA correlator. Then the same data set was fed to the design under test and results after second stage integration were saved in a file. Again using MATLAB, the results from MATLAB and RTL functional simulation were compared to evaluate the correctness of our design.

**Figure 5:**
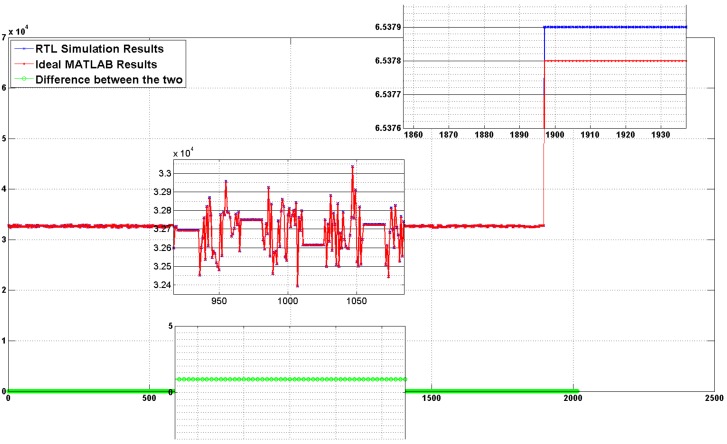
Comparing MATLAB and RTL simulation results and showing the difference.

Results from Vivado RTL simulation exactly followed the results from MATLAB verifying the functionality of our design. The plot of difference between both the results is a straight line showing a constant bias of 1 in RTL design, as shown in Figure 5. Even this difference of one is because of first pipeline stage, as it takes one clock cycle after power-on reset for data to reach the input of correlator. At power-on reset all the registers, including first pipeline stage registers that latch the inputs, are at the same level so for correlator the first sample is all one or all zeroes adding one false correlation to overall result. Other than this difference, both the plots exactly follow each other throughout, as obvious from zoomed in parts of Figure 5.

The design was synthesized and implemented in Vivado platform with some timing constraints. The high-speed clock was constrained to run at 3.8 ns (~263 MHz) and the slow-speed clock to be exactly 6 time slower i.e., 22.8 ns (~43 MHz). Implementation results show that these constraints were successfully met for all paths in the design. It can safely be assumed that this correlator can perform well at 250 MHz resulting in a collective capacity of 2 GHz from all 8 lanes. This is where our proposed design partition technique for correlator implementation proved fruitful, as our first design without this partition could only run at 200 MHz giving a total of 1.6 GHz throughput as described in [26]. Device view shows that the correlation and first stage integration modules, running at higher frequency, were implemented using resources with minimum physical separation while the modules containing second stage of integration were implemented using device resources scattered over comparatively large area owing to the fixed location of BRAM and DSP slices. However, the second stage integration could easily meet timing requirements as it was clocked by the slower signal.

Device utilization is reflected in the Table 1. It is quite obvious that approximately 50% of the device is utilized. It would enable the digitization circuit interface and master processor interface to be implemented on the same device. In this way, all the digital correlation system for 64-channel, except A/D front-end circuit would be implemented on a single chip reducing inter-chip communication overhead and component count in the system. Device utilization is where we have paid the price of achieving higher throughput by consuming considerably more hardware resources on-chip. It can be observed in Table 1 that while only 2% more device look-up tables are utilized, the utilization of registers is almost 50% more than our baseline implementation, approaching to 51% of total available registers in the device. However, that still leaves quite a lot of resources for interface and control circuitry to be implemented on the same device.

Power consumption estimate in Table 2, as provided by the Vivado, reflects a total of 7.457 W for the operation of this design at 263 MHz. This is equivalent to 1.76 mW/correlator/GHz, 9–14 times higher than the ASIC but 4–8 times less than the FPGA implementation as reported by [19]. This is partly because of newer device used for our implementation and partly because of keeping high speed processing localized and performing all other possible operations at lower speed. Although FPGA is not a very suitable platform for measuring power efficiency because of its configurable nature and circuit implementation methodology, our results shows some power saving as compared to baseline implementation as per expectations. Our design partitioning has paid off on this front as well by consuming lower power due to localization of high frequency circuit and letting a considerable part of the circuit to run at lower frequency.

## 5. Conclusions

Design of a digital correlator for Passive Millimeter Wave Imager is presented in detail with a focus on improving throughput using frequency based design partitioning. This correlator is designed for a system using SAIR technique, therefore, the presented design can also find its application in other remote sensing applications based on this imaging method. Total throughput achievable by this design on Xilinx Kintex UltraScale XCKU115 device approaches to 4 Trillion 1B/2L correlation and accumulation operations per second as a result of running 8 lanes of 64-channel correlators in parallel at 250 MHz. The results obtained in simulation exactly follow the expected results as calculated in MATLAB, verifying the functional correctness. Synthesis and implementation results from Vivado platform proves the throughput claims and feasibility of implementation on the mentioned device. Comparison of results with our baseline implementation shows (Table 1 and Table 2) the gains and cost (in terms of hardware utilization) of using frequency based design partitioning at logic level for a digital cross correlator.

Overall, this correlator design fits well in imaging system, meeting all functional and practical requirements. However, we currently focus on reducing device utilization that has increased substantially as a result of applying design partitioning technique. Reducing hardware requirements can allow using smaller devices for imaging system based on the combination of aperture synthesis and analog phased array techniques and save a handsome amount of financial resources effectively reducing the system cost. On the other hand, reduction in device utilization can increase the maximum number of correlation channels in a single device which is the key factor deciding the maximum achievable scale for our 2D aperture synthesis system.

## Figures and Tables

**Table 1:** Device utilization for 64-channel correlator using KCU115.

Resource	Available	With Partition (2 Gbps/Channel)		Without Partition (1.6 Gbps/Channel)	
		Used	Utilization %	Used	Utilization %
CLB LUTs	663,360	282,898	42.65	266,975	40.25
CLB Registers	1,326,720	679,654	51.23	460,158	34.68
CARRY8	82,920	32,256	38.90	32,256	38.90
F7 MUXes	331,680	26,616	8.02	26,520	8.00
F8 MUXes	165,840	3468	2.09	3852	2.32
Block RAM Tiles	2160	64	2.96	64	2.96
DSPs	5520	64	1.16	64	1.16

**Table 2:** Device power estimates for 64-channel correlator using KCU115.

Parameter	With Partition (2 Gbps/Channel)	Without Partition (1.6 Gbps/Channel)
Total On-Chip Power (W)	7.457	8.050
Dynamic Power (W)	5.350	5.842
Device Static Power (W)	2.107	2.208
Effective TJA (°C/W)	3.8	3.8
Max Ambient (°C)	56.7	54.4
Junction Temperature (°C)	58.3	60.6
Confidence Level	High	High
